# Regulation of Osteosclerosis by Inoculated Cd133^+^ PC3 Cells in Bone‐marrow Microenvironmental Niches

**DOI:** 10.1002/jbm4.10189

**Published:** 2019-05-20

**Authors:** Donghwi Kim, Youngjong Ko, Mineon Park, Bora Kim, HongMoon Sohn, Wonbong Lim

**Affiliations:** ^1^ Department of Orthopaedic Surgery; ^2^ Laboratory of Orthopaedic Research Chosun University Hospital Gwangju South Korea; ^3^ Department of Premedical Program School of Medicine, Chosun University Gwangju South Korea

**Keywords:** Bone marrow stromal cell, Cancer stem cell, CD133, Microenvironmental niche, Osteosclerosis

## Abstract

Bone is the most common site of prostate cancer (PC) metastasis. Studies suggest that cancer stem cells (CSCs) are associated with stemness characteristics, providing some support for the concept that CSCs act as osteosclerotic precursors in bone microenvironmental niches. Here, we asked whether ectopic overexpression of CD133 maintains stability of CSCs in human PC cell lines and induces the changes of molecular features in the bone microenvironment. Ectopic overexpression of CD133 in PC3 or DU145 cells led to increased expression of ALDHA1, OCT4, and NANOG, enhanced colony‐forming ability, and increased ALDH activity. In addition, micro‐CT imaging, confocal microscopy, and H&E staining of mouse tissue confirmed that CD133 overexpression in PC3 and DU145 led to marked osteolytic bone tumor. However, expression of osteoblastic markers such as collagen type I, bone sialoprotein, and osteocalcin (OC) at the tumor margin of CD133‐overexpressing PC3 tumors in mouse tibiae was higher than that of CD133‐overexpressing DU145 tumors with osteosclerotic molecular features. In addition, expression of osteopontin (OPN) mRNA/protein by CD133‐overexpressing PC3 cells was higher than that by DU145 cells. Especially, conditioned medium (CM) from PC3^CD133+^ cells increased osterix (OSX) activity in bone marrow stromal cells (BMSCs), resulting in increased expression of OC mRNA/protein resulted in increased staining of mineralized matrix by Alizarin red. However, CM from OPN silenced PC3^CD133+^ cells led to a reduction of OC mRNA and protein expression through OSX activity resulted in reduced amount of mineralized matrix. In conclusion, these findings suggest that CD133 plays a functional role in regulating CSC characteristics in PCs and modulates their abilities in which induce the osteosclerosis of BMSCs. In addition, OPN from CSCs acts as a niche component that promotes osteosclerosis by supporting osteoblastic differentiation of BMSCs. © 2019 The Authors *JBMR Plus* published by Wiley Periodicals, Inc. on behalf of American Society for Bone and Mineral Research.

## Introduction

About 90% patients with advanced prostate cancer (PC) have incurable bone metastases, with a mean survival of 1 year.^1^, ^2^ Bone metastasis results in significant complications, including bone pain, impaired mobility, pathological fracture, spinal cord compression, and symptomatic hypercalcemia.^3^, ^4^ In particular, the osteosclerotic nature of most bone metastases related to PC means that there is a marked increase in osteoblastic activity.^5^ There is, however, clear histological evidence of increased bone resorption in these patients, even in the absence of overt osteolytic bone metastases.^6^, ^7^ Therefore, a better understanding of the early mechanisms underlying development of bone metastasis would allow therapies to be specifically targeted to early lesions as they arise.

The hypothesis that tumors depend on a small fraction of cells, called cancer stem cells (CSCs), for long‐term survival is based on data demonstrating that subsets of human leukemic cells transferred tumor‐initiating activity to SCID mice.^8^ Recent studies show that PC stem‐like cells originating from a primary tumor have a high potential for invasion and possess self‐renewal and clonogenic properties.^9^ These cells can be identified by expression of stem cell markers such as aldehyde dehydrogenase 1 (ALDH1), CD133, c‐Met, and prostate stem cell antigen.^10^ It is considered that CSCs are typically dormant and that their later regrowth triggers metastasis. Also, the molecular machinery of the stem cell niches is designed to regulate stem cell quiescence and self‐renewal; therefore, we hypothesized that CSC‐like cells may alter the osteoblastic or osteoclastic progenitor stem cell phenotype via engagement with the niche, thereby establishing a future site of metastasis.

Here, we used murine models, in which PC cells would be inoculated in tibial bone marrow for establishment of interaction with bone marrow stromal cells (BMSCs) and metastatic PCs.^11^ It will show that CSC‐like cells affect the destination of pre‐osteoblasts and pre‐osteoclasts, which are significantly enriched for a mesenchymal and hematopoietic stem cell phenotype in the bone marrow.^12^ CSCs in the bone marrow were maintained over time, a phenomenon not caused by effects on proliferation, homing, or cell survival in the circulation. Differentiation of osteocytes and osteoclasts occurred in the bone marrow following injection of CSC‐like cells, resulting in establishment of metastases in the marrow. However, it is unclear which CSC characteristics affect differentiation of osteocytes or osteoclasts.

CD133 (prominin‐1), a 5‐transmembrane glycoprotein, was first identified as a marker of hematopoietic stem cells.^13^ Consequently, CD133 is considered an important cell surface marker for a subpopulation of cancer initiating cells (CICs) in brain tumors, colon carcinoma, head and neck cancer, hepatocellular carcinoma, thyroid carcinoma, and PC.^14^, ^15^, ^16^, ^17^ Previously, we demonstrated that upregulation of CD133 on head and neck cancer cells and in head and neck squamous cell carcinoma tissue correlates with chemoresistance to anticancer drugs such as 5‐FU.^18^, ^19^ Recent reports suggest that expression of CD133 by tumor tissues may be a prognostic indicator for tumor regrowth, malignant progression, and patient survival.^20^ Nevertheless, the CD133‐mediated molecular mechanisms that regulate differentiation of osteocytes and osteoclasts during bone metastasis of PC remain unclear.

The hypothesis presented herein show that CD133 in PCs plays a critical role in increasing stemness, thereby promoting formation of aggressive tumors in murine model when the cells were inoculated in tibial bone marrow. Double transfection of PC cells with green fluorescence protein (GFP) and DsRed2 revealed the distribution of CD133‐overexpressing PC cells in the bone marrow. In addition, we tried to identify which niche components secreted by CD133 + PC play a critical role in osteoblastic differentiation of bone marrow–derived stromal cells (BMSC). Ultimately, we demonstrate that CD133 + PC play a critical role in bone remodeling during the progression of PC’s bone metastasis.

## Materials and Methods

### 2.1 Cell culture

PC3, DU145, HT29 and 293 T cells were purchased in 2017, from the Korean Cell Line Bank (KCLB; no. 21435, 30081, 30038 and 21573) Korean Cell Line Research Foundation, Seoul, Korea. The cells were authenticated under short tandem repeat analysis by the cell bank. And then the cells were expanded and stored in liquid nitrogen upon receipt, and each aliquot was passaged for fewer than 6 months in our laboratory. The cells were maintained at 37°C/5% CO_2_ in RPMI 1640 (Welgene, Daegu, Korea) supplemented with 10% heat‐inactivated fetal bovine serum (FBS; Gibco BRL, Grand Island, NY, USA) and 0.1% antibiotic/antimycotic solution (Welgene). After seeding, the medium was replaced with fresh medium and adherent cells were allowed to reach about 70% confluence. The cells were then detached with trypsin/EDTA (Gibco BRL) and replated (subcultured) in 6‐well plates for each experiment.

### 2.2 Cloning of human CD133

HT29 colon cancer cells were used as a source of CD133. Cloning of CD133 was carried out as described previously.^18^ Plasmid pcDNA3.1/NT‐GFP lacking the CD133 insert was used as a transfection control. To check for the translated fusion product, 293 T cells were used as a positive control. Briefly, 293 T cells grown to near‐confluence in a 60 mm dish were transfected transiently with pcDNA3.1/NT‐GFP or pcDNA3.1/NTGFPCD133 (Promega, Madison, WI, USA; ratio = 0.2 μg of plasmid to 0.6 μl of reagent) using the FuGENE HD transfection reagent (Promega). After 48 hours, cells were gently washed with ice‐cold phosphate‐buffered saline (PBS) and lysed.

### 2.3 Establishment of stably transfected PC3‐GFP/DU145‐GFP control cells and PC3^GFP‐CD133^/DU145^GFP‐CD133^ cells expressing the fusion protein

Stable cell lines overexpressing GFP and the CD133‐GFP fusion protein were created by transfecting confluent PC3 or DU145 cells in 100 mm plates with 20 μg of pcDNA3.1/NT‐GFP::CD133 or pcDNA3.1/NT‐GFP plasmid using the FuGENE HD transfection reagent as described previously.^18^ Briefly, cells stably expressing GFP‐CD133 or GFP were selected using 0.4 mg/mL G‐418 sulfate (A.G. Scientific INC., San Diego, CA, USA) in DMEM containing 4 mM glutamine, 1 mM pyruvate, 4.5 g/L glucose, and 10% FBS. After transfection, surviving green fluorescent cells expressing various levels of CD133‐GFP or GFP were expanded into 60 mm wells. Stably transfected cells were further expanded and passaged onto 100 mm tissue culture plates using 0.05% trypsin/0.2 g/L EDTA/4Na in Hanks Balanced Salt Solution (Welgene) and cultured with 2 mg/mL G‐418 to maintain expression of GFP‐CD133 or GFP.

### 2.4 Western blot analysis

When cells reached about 70% confluence, the medium was removed and they were washed twice with PBS (pH 7.4). Next, cell lysates were prepared in 200 ml of cold lysis buffer (1% NP‐40, 50 mM Tris‐HCl, pH 7.5, 150 mM NaCl, 0.02% sodium azide, 150 mg/mL PMSF, 2 mg/mL aprotinin, 20 mg/mL leupeptin, and 1 mg/mL pepstatin A). Approximately 30 mg of tissue lysate was separated by 10% sodium dodecyl sulfate polyacrylamide gel electrophoresis and transferred to a polyvinylidene difluoride membrane (Amersham, Piscataway, NJ, USA). Each membrane was blocked for 0.5 hours with a blocking solution containing 5% skim milk in Tris‐buffered saline‐Tween (TBST; 2.42 g/L Tris‐HCl, 8 g/L NaCl, 0.1% Tween 20, pH 7.6) and rinsed briefly in TBST. The membrane was incubated overnight at 4°C with the appropriate primary antibody: anti‐CD133 (1:1000; MyBioSource, San Diego, CA, USA), anti‐OCT‐4 (1:1000; Cell Signaling Technology, Beverly, MA, USA), anti‐ALDHA1 (1:1000; Cell Signaling Technology), anti‐NANOG (1:1000; Cell Signaling Technology), anti‐DsRed2 (1:1000; Santa‐Cruz Biotechnology, Santa‐Cruz, CA, USA), anti‐OPN (1:1000; Cell Signaling Technology), anti‐OC (1:1000; Cell Signaling Technology), anti‐Runx2 (1:1000; Cell Signaling Technology), or anti‐OSX (1:1000; Cell Signaling Technology). A mouse monoclonal IgG specific for GAPDH (1:2500; Santa‐Cruz Biotechnology), α‐tubulin (1:2500; Santa‐Cruz Biotechnology), or β‐actin (1:2500; Santa‐Cruz Biotechnology) was used as a control. Finally, the membrane was washed in TBST and the immunoreactivity of the proteins was detected using an enhanced chemiluminescence detection kit (Amersham).

### 2.5 Confocal microscopic analyses

Cells were counterstained with 4′,6‐diamidino‐2‐phenylindole provided in ProLong Gold antifade mounting medium (Invitrogen, Carlsbad, CA, USA) to visualize nuclear morphology. Digital images were captured at the Korea Basic Science Institute Gwangju Center using a TCS SP5 AOBS laser‐scanning confocal microscope (Leica Microsystems, Heidelberg, Germany) fitted with a × 20 objective lens.

### 2.6 Colony‐forming assays

Cells were seeded at a density of 1000 cells per well in nonadherent 24‐well culture plates coated with a 10% polyHEMA (Sigma‐Aldrich, St. louis, MO, USA) solution in absolute ethanol and then dried overnight. After seeding, cells were incubated in a serum‐free DMEM‐F12 medium supplemented with 200 ng/mL EGF (Sigma‐Aldrich), 20 ng/mL basic FGF (Sigma‐Aldrich), and B‐27 supplement (Invitrogen). After 5 days of incubation, the number of spheroids in each well was counted under a light microscope (Zeiss, Zena, Germany).

### 2.7 Flow cytometric assessment of ALDH activity

This assay of tumor‐derived cells was conducted using the ALDEFLUOR kit (Stem Cell Technologies, Seattle, WA, USA). Cells were suspended in ALDEFLUOR assay buffer containing ALDH1 substrate (BODIPY‐aminoacetaldehyde, 1 M per 10^6^ cells) and incubated for 30 minutes at 37°C. As a negative control, some batches of cells were incubated with a specific ALDH1 inhibitor, diethylaminobenzaldehyde (DEAB; 50 mM). Next, cells were washed twice with washing buffer and analyzed by flow cytometry (Beckman Coulter, CA, USA). Approximately 10,000 events were captured per sample.

### 2.8 Cloning of DsRed2 protein and establishment of stably transfected PC3^DsRed2^/DU145^DsRed2^ control cells and PC3^DsRed2GFP‐CD133^/DU145^DsRed2GFP‐CD133^ cells expressing the fusion protein

To monitor the distribution of injected cells, wild‐type or GFP‐CD133 + cells were transfected with DsRed2. In brief, PC3/DU145 and PC3‐GFP‐CD133/DU145‐GFP‐CD133 cells stably expressing DsRed2 were selected using 10 mg/L Blasticidin (Invivogen, San Diego, CA, USA) in RPMI1640 containing 4 mM glutamine, 1 mM pyruvate, 4.5 g/L glucose, and 10% FBS. After transfection, surviving red fluorescent cells expressing various levels of DsRed2 were expanded into 60 mm wells. Stably transfected cells were further expanded and passaged onto 100 mm tissue culture plates using 0.05% trypsin/0.2 g/L EDTA•4Na in Hank's Balanced Salt Solution (Welgene) and cultured with 10 mg/L Blasticidin to maintain expression of DsRed2 protein. Images of intracellular DsRed2 and GFP fluorescence were obtained by confocal microscopy (Leica Microsystems).

### 2.9 Animals

Five‐week‐old male athymic nude mice (BL‐6/Nu, Orient Bio Co. LTD, Seoul, Korea) were housed under controlled light conditions and fed ad libitum. All experimental procedures involving animals were performed in compliance with institutional and governmental requirements and were approved by the Institutional Animal Care and Use Committee (CIACUC2015‐A0032), Chosun University, Gwangju, Korea.

### 2.10 Intra‐tibial xenograft model

Intra‐tibial injection of PC cells was used to examine the ability of tumor formation in the bone marrow. The mice were anesthetized with isoflurane gas and then, PC3^DsRed2+^, PC3^DsRed2+CD133+^, DU145^DsRed2+^, or DU145^DsRed2+CD133+^ cells (1 × 10^5^ per mouse) were injected into the right tibia. Briefly explained, both knee joints were shaved and cells (in 20 μl of DMEM) were injected using a 100 μl Hamilton‐type syringe with a 27 gauge needle. The needle was inserted into the intra‐condylar notch of the femur using a spinning motion. Cells were injected into the medullary space (approximately 0.3 cm proximal to the epiphyseal plate). To control for surgical effects, 5 μl of DMEM was injected into the contralateral limb. Following injection, 0.1 mg/kg buprenorphine was injected subcutaneously to minimize postprocedure pain and animals were returned to cages for recovery. Tumor‐bearing tissues from mice at necropsy were obtained 4 weeks later.

### 2.11 Histologic analysis of mouse tissues

Tumor‐bearing tissues were fixed in cold 4% paraformaldehyde. Bone tissue was first decalcified in sodium citrate solution before processing into histologic slides. Decalcified bones were cut at the midpoint and embedded in paraffin blocks. The fluorescence in serial paraffin sections was monitored by fluorescence microscopy (Leica Microsystems). Tissues were stained with H&E, and images were captured using a microscope slide scanner (3D‐HISTECH Ltd., Budapest, Hungary).

### 2.12 Micro‐CT analysis

The right femur was dissected, and CT imaging was performed using a Quantum GX μCT imaging system (PerkinElmer, Hopkinton, MA, USA) at the Korea Basic Science Institute (Gwangju, Korea). The X‐ray source was set at 90 kV/88 mA, with a field of view of 45 mm (voxel size, 90 μm; scanning time, 14 minutes). The CT imaging was presented in the 3D Viewer within Quantum GX. Following scanning, image segmentation was performed using Analyze software (AnalyzeDirect, Overland Park, KS, USA). Briefly, segmentation of the leg was performed using both semi‐automatic and manual tools; object extraction, region growing, and objector separator were performed using the Volume Edit tool. Subsequently, a 3D rendering of the leg and tumor was generated and tumor volumes were calculated using the ROI tool.

### 2.13 Immunohistochemical analysis of bone specimens

Sections (Results μM thick) were deparaffinized in three changes of xylene and rehydrated in a graded series of ethanol solutions (ending with distilled water). For antigen retrieval, slides were placed in 0.01 M citrate‐buffer, pH 6.0, and heated in a steamer for 30 minutes. Endogenous peroxidases were quenched by incubating with 3% hydrogen peroxide for 20 minutes at room temperature. Sections were incubated overnight at 4°C with a 1:50 dilution of primary antibody: anti‐collagen Col1 (Santa‐Cruz Biotechnology), anti‐BSP (Santa‐Cruz Biotechnology), anti‐OC (Santa‐Cruz Biotechnology), or anti‐NFATC1 (Santa‐Cruz Biotechnology). Subsequently, sections were incubated for 30 minutes with a biotinylated secondary antibody (LSAB; Dako Cytomation, Glostrup, Denmark), washed in PBS, and incubated for 30 minutes with a streptavidin‐peroxidase conjugate (LSAB, Dako Cytomation). The reaction was developed for 5 minutes using 3,30‐diaminobenzidine tetrahydrochloride (Sigma‐Aldrich). Slides were briefly counterstained in hematoxylin, dehydrated, and cover slipped. Negative and positive controls were run simultaneously. Positive controls comprised mammary tissue. The slides were captured using a microscope slide scanner (3D‐HISTECH Ltd.).

### 2.14 Enzyme‐linked immunosorbent assay

The amount of Receptor activator of nuclear factor kappa‐Β ligand (RANKL) in cell culture supernatants and serum of xenograft‐bearing mice was measured using a commercially available enzyme immunoassay kit (R&D Systems, MN, USA), according to the manufacturer's protocol. Absorbance was measured at 450 nm in a colorimetric microplate reader (BioTek, Winooski, USA). The reading was then subtracted from the reading at 570 nm.

### 2.15 Cytokine profiling

Supernatants from PC cells were collected and assayed using a human inflammation antibody array (R&D Systems), according to the instruction manual. Table 1 lists the cytokines examined using this technique. The cytokine signal was detected using an ECL detection kit (Amersham) and quantified by densitometric analysis using Image J software (Scion Corp, MD, USA). The signal intensity of each spot was adjusted to that of the corresponding internal control provided for each membrane by the manufacturer (cytokine profiling). The ratio of expression was calculated by dividing the signal intensity of each cytokine by the signal intensity of the internal control for each sample.

**Table 1:** Characteristics of the Intra‐tibial Tumors. *P* Values (Chi Squared Test) were Used to Compare Tumor Occurrence or Invasion after Injection of PC3^DsRed2+CD133+^ or PC3^DsRed2+^, or DU145^DsRed2+CD133+^ or DU145^DsRed2^, Cells

Cell lines	PC3^*DsRed2+*^	PC3^*D/sRed2+GFPCD13*3+^	DU145^*DsRed*2+^	DU145^*DsRed2+GFPCD133+*^
Tumor occurrence % (Number of mice with tumors/mice in study)	3/10 (30%)	10/10 (100%), *P* < 0.05	0/10 (0%)	4/10 (40%)
Tumor invasion outside bone marrow cavity % (Number of mice with invading tumor/mice in study)	2/10 (20%)	10/10 (100%), *P* < 0.05	0/10 (0%)	2/10 (20%)

### 2.16 Culture of osteoblast progenitors

Osteoblast progenitor cells were isolated from bone marrow by flushing the tibiae and femurs with a‐minimal Essential Medium (MEM) medium (Invitrogen), as previously described.^21^ After red blood cells were depleted with ACK (ammonium‐chloride‐potassium) lysis buffer (0.01 mM EDTA, 0.011 M KHCO_3_, and 0.155 M NH_4_Cl, pH 7.3), the remaining cells were suspended in complete a‐MEM supplemented with 10% (v/v) FBS, 100 U/mL penicillin, and 100 mg/mL streptomycin. The plastic‐adherent fibroblast‐like cells (so‐called BMSCs; approximately 80–90% confluence) were subcultured using 0.25% trypsin‐EDTA (Gibco BRL) and replated at a density of 1 × 10^4^ cells/cm^2^ for further expansion.

### 2.17 Coculture assays

CM was collected from PC cells. The medium was harvested, sterile filtered, and stored at − 20°C until needed. For coculture assays, BMSCs were seeded at a density of 5 × 10^4^/cm^2^ and cultured in 10% α‐MEM supplemented with 50% (v/v) CM in the presence of osteoblastic inducers (50 mg/mL ascorbic acid;AA and 5 mM β‐Glycophosphate;β‐GP). The medium was replaced every 48 hours, and differentiation was examined at the indicated times.

### 2.18 siRNA‐mediated knockdown of OPN

PC or BMSCs were plated in 6‐well plates (2 × 10^5^ cells per well) for 24 hours. The medium was removed, and cells were transfected with 30 nM control/hOPN1/hOPN2 siRNA oligonucleotide duplexes (for PC) or control/mOPN1/mOPN2 siRNA (for BMSCs) using the transfection reagent according to the manufacturer's instructions (Qiagen, Valencia, CA, USA). The cells were then incubated at 37°C/5% CO_2_ in medium lacking antimicrobial agents for 48 hours. Next, siRNAs were removed and the medium was replaced by fresh medium for 24 hours. Irrelevant control siRNA (nonspecific control) was purchased from Dharmacon/Thermo Fisher Scientific (Lafayette, CO, USA). The hOPN1 sequence used for targeted RNA interference was 5′‐CUUCUGAGAUGGGUCAGGGTT‐3′, and the hOPN2 sequence was 5′‐UUUCGUUGGACUUACUUGGTT‐3′.^22^ The mOPN1 sequence used for targeted RNA interference was 5′‐GCUUUACAGCCUGCACCCATT‐3′, and the mOPN2 sequence was 5′‐GCCAUGACCACAUGGACGATT‐3′.^23^

### 2.19 Alizarin red staining

Cells were fixed in 95% ethanol and treated for 30 minutes with 40 mM Alizarin red stain (AR‐S) solution (pH 4.2) to label calcium deposits. Stained cultures were photographed, and the AR‐S was extracted with 10% (w/v) cetylpyridinium chloride in 10 mM sodium phosphate (pH 7.0). The AR‐S concentration was determined by measuring the absorbance at 540 nm and reading off an AR‐S standard curve.

### 2.20 ALP assay

The cellular proteins were solubilized with 1% Triton X‐100 in 0.9% NaCl and centrifuged. The cell homogenates were reacted with the ALP assay mixture containing 0.1 M 2‐amino‐2‐methyl‐1‐propanol (Sigma), 1 mM MgCl_2_, and 8 mM p‐nitrophenyl phosphate. After 5 minutes of incubation at 37℃, the reaction was quenched by adding 0.1 N NaOH and the absorbance was measured at 405 nm. Protein concentrations were measured using a Protein Assay Kit (Bio‐Rad, CA, USA), and ALP activity was normalized to cellular protein content.

### 2.21 Quantitative real‐time PCR analysis

Total RNA was extracted from cells using Trizol (Invitrogen). Complementary DNA (cDNA) was synthesized from 2 μg of total RNA using the Super‐Script II First‐Strand Synthesis System (Invitrogen). mRNA levels were measured using quantitative real‐time PCR. The glyceraldehyde‐3‐phosphate dehydrogenase (*Gapdh*) gene was used as an endogenous control. The sequences of the primers used to target various genes are shown in Table 2. Details of the reaction system and amplification procedures have been described previously.^24^

**Table 2:** List of Human Inflammatory Cytokines Examined Using the Antibody Array (R&D Systems)

Coordinate	Analyte/Control	Entrez Gene ID	Alternate Nomenclature
A1, A2	Reference Spots	N/A	RS
A3, A4	Adiponectin	9370	Acrp30
A5, A6	Apolipoprotein A‐I	335	ApoA1
A7, A8	Angiogenin	283	______
A9, A10	Angiopoietin‐1	284	Ang‐1, ANGPT1
A11, A12	Angiopoietin‐2	285	Ang‐2, ANGPT2
A13, A14	BAFF	10673	BLyS, TNFSF13B
A15, A16	BDNF	627	Brain‐derived Neurotrophic Factor
A17, A18	Complement Component C5/C5a	727	C5/C5a
A19, A20	CD14	929	______
A21, A22	CD30	943	TNFRSF8
A23, A24	Reference Spots	N/A	RS
B3, B4	CD40 ligand	959	CD40L, TNFSF5, CD154, TRAP
B5, B6	Chitinase 3‐like 1	1116	CHI3L1, YKL‐40
B7, B8	Complement Factor D	1675	Adipsin, CFD
B9, B10	C‐Reactive Protein	1401	CRP
B11, B12	Cripto‐1	6997	Teratocarcinoma‐derived Growth Factor
B13, B14	Cystatin C	1471	CST3, ARMD11
B15, B16	Dkk‐1	22943	Dickkopf‐1
B17, B18	DPPIV	1803	CD26, DPP4, Dipeptidyl‐peptidase IV
B19, B20	EGF	1950	Epidermal Growth Factor
B21, B22	Emmprin	682	CD147, Basigin
C3, C4	ENA‐78	6374	CXCL5
C5, C6	Endoglin	2022	CD105, ENG
C7, C8	Fas Ligand	356	TNFSF6, CD178, CD95L
C9, C10	FGF basic	2247	FGF‐2
C11, C12	FGF‐7	2252	KGF
C13, C14	FGF‐19	9965	______
C15, C16	Flt‐3 Ligand	2323	FLT3LG
C17, C18	G‐CSF	1440	CSF3
C19, C20	GDF‐15	9518	MIC‐1
C21, C22	GM‐CSF	1437	CSF2
D1, D2	GROα	2919	CXCL1, MSGA‐α
D3, D4	Growth Hormone	2688	GH, Somatotropin
D5, D6	HGF	3082	Scatter Factor, SF
D7, D8	ICAM‐1	3383	CD54
D9, D10	IFN‐γ	3458	IFNG
D11, D12	IGFBP‐2	3485	______

### 2.22 Luciferase reporter assay

BMSCs were transiently transfected with a Runx2 or OSX target sequence‐linked luciferase reporter plasmid using Lipofectamine (Invitrogen), according to the manufacturer's instructions. After transfection, cells were incubated in the designated medium for 7 days. Luciferase activity was measured using the Luciferase Assay System (Promega) and detected using a luminometer (GENios Plus; Tecan Group Ltd., Salzburg, Austria). The pCMV‐β‐gal expression vector was added to each transfection, and the β‐galactosidase assay was carried out as described previously to normalize transfection efficiency.^25^

### 2.23 Statistical analysis

A two‐tailed, paired the student t test and and one‐way ANOVA followed by Sidak's multiple comparison test (unless speciﬁcally mentioned otherwise) when comparing more than two groups were used for statistical analyses. A *P* value < 0.05 was considered statistically significant. Data are expressed as the mean ± standard deviation (SD) unless specified otherwise and all experiments were performed in triplicate. The GraphPad Prism version 6.00 software program for Windows (GraphPad, La Jolla, CA, USA) was used to analyze data from in vitro and in vivo experiments.

## Results

### 3.1 Stable overexpression of CD133 in PC3 and DU145 cell lines

**Figure 1:**
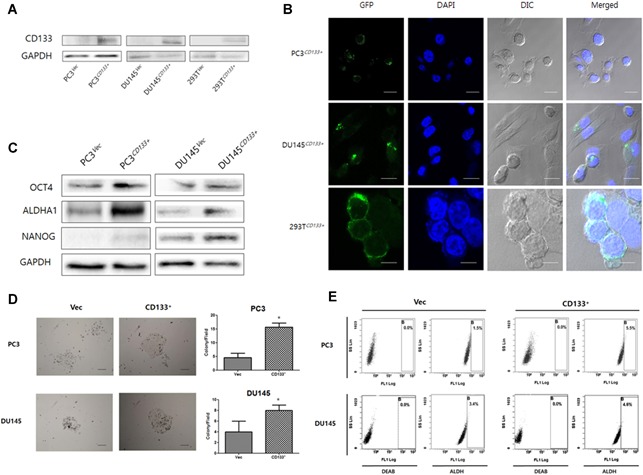
Overexpression of CD133 in prostate cancer cell lines. *A*, Western blot analysis of CD133 expression by PC3^Vec^/DU145^Vec^ (controls) and PC3^CD133+^/DU145^CD133+^ (CD133‐overexpressing) cells. 293 T cells transiently transfected with pcDNA3.1/NT‐GFP or pcDNA3.1/NTGFPCD133. 293 T^Vec^/293 T^CD133+^ cells were used as a positive control. *B*, Confocal microscopy analysis of GFP‐CD133 expression by stably or transiently transfected PC3/DU145/293T‐GFP‐CD133 cells. Nuclei in both sets of images are stained with DAPI (blue). Images were taken at 630 × magnification. Bar, 10 μm. *C*, Western blot analysis of expression of stemness‐related proteins. GAPDH was used as a loading control. *D*, Stable overexpression of CD133 led to a significant increase in colony‐forming ability by PC3 cells and a slight increase in ability by DU145 cells. Images were taken at 100 × magnification. Bar, 100 μm. Bar graphs showing the mean ± the standard deviation (SD). *, *P* < 0.05. *E*, Representative flow cytometry analyses of ALDH activity. The first two columns on the left represent negative controls (cells treated with the ALDH inhibitor diethylaminobenzaldehyde (DEAB). The two columns on the right represent cells in the B gate (the ALDH + population).

To determine the effect of CD133 overexpression in vitro, we first established stable cell lines overexpressing CD133 under the control of a constitutive promoter. Specifically, we transfected the PC cell lines PC3 and DU145 with either GFP‐tagged CD133 or an empty vector containing GFP only. A representative CD133‐GFP clone was selected from each line (PC3^CD133+^ or DU145^CD133+^) and then compared with a control cell line transfected with the empty vector (PC3^Vec^ or DU145^Vec^). 293 T cells transiently transfected with GFP (293 T^Vec^) or CD133‐GFP (293 T^CD133+^) were used as a positive control. Basal expression of CD133 protein by PC3^Vec^ and DU145^Vec^ cells was very low; however, that by stable CD133‐GFP–transfected cells (PC3^CD133+^ and DU145^CD133+^) and transiently transfected 293T cells (Fig. 1 *A*) was apparently higher. Green fluorescence was detected in the cytosol and on the cell membrane of PC3^CD133+^ and DU145^CD133+^ cells (Fig. 1 *B*).

Next, to investigate the role of CD133 in cancer stemness we examined acquisition of CSC‐like properties, such as an increased ability to form tumor spheres, increased ALDH activity, and increased expression of stem cell‐like markers such as ALDHA1, OCT4, and NANOG. Expression of OCT4, ALDHA1, and NANOG proteins by PC3^CD133+^ and DU145^CD133+^ increased apparently, consistent with increases in properties associated with stemness (Fig. 1 *C*). Stemness was also confirmed in a colony‐formation assay. PC3^CD133+^ and DU145^CD133+^ showed an enhanced ability to form colonies, a property concordant with increased expression of stemness markers (Fig. 1 *D*).

Finally, we used an ALDEFLUOR assay to examine the presence and size of the PC3^CD133+^ and DU145^CD133+^ cell populations exhibiting enzyme activity (Fig. 1 *E*). Of the PC3^Vec^ and DU145^Vec^ cells examined, 1.5% and 3.4%, respectively, were ALDEFLUOR‐positive. By contrast, 5.5% and 4.6% of PC3^CD133+^ and DU145^CD133+^ cells, respectively, were ALDEFLUOR‐positive. These results demonstrate that ectopic overexpression of CD133 promotes stem‐like properties, including increased ALDH activity, increased colony‐forming ability, and increased expression of ALDHA1, OCT4, and NANOG.

### 3.2 Stable overexpression of DsRed2 in PC3 and DU145

**Figure 2:**
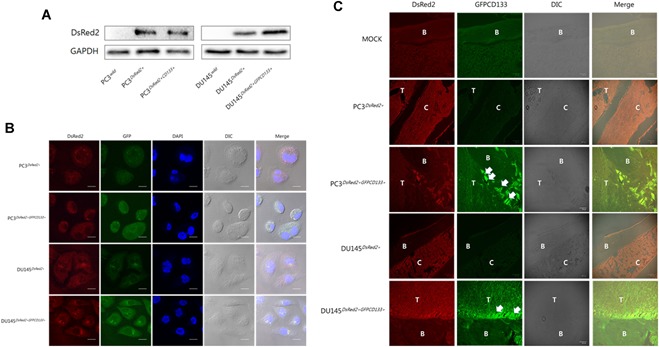
Plasmids expressing the indicated fluorescence proteins were used for double transfection of prostate cancer cell lines. *A* Western blot analysis of DsRed2 expression in PC3^wild^/PC3^DsRed2+^/PC3^DsRed2+CD133+^ and DU145^wild^/DU145^DsRed2+^/DU145^DsRed2+CD133+^ cells. *B*, Confocal microscopy of DsRed2 expression by stably transfected PC3^DsRed2+^/PC3^DsRed2+CD133+^ and DU145^DsRed2+^/DU145^DsRed2+CD133+^ cells. Nuclei were stained with DAPI (blue). Images were taken at 630 × magnification. Bar, 10 μm. *C*, Immunofluorescence of GFP and DsRed2 in the tibiae of mice at 28 days following injection of PC3^DsRed2+^/PC3^DsRed2+CD133+^ and DU145^DsRed2+^/DU145^DsRed2+CD133+^ cells. Images were taken at 100 × magnification. Bar, 100 μm. B, Bone; T, Tumor mass; C, Injected cancer cell.

To enable monitoring of cell distribution after injection into mouse tibiae, PC3^wild^/PC3^CD133+^ and DU145^wild^/DU145^CD133+^ cells were transfected with DsRed2. Expression of DsRed2 was higher in stably transfected cells (PC3^DsRed2+^/PC3^DsRed2+GFP‐CD133+^ and DU145^DsRed2+^/DU145^DsRed2+GFP‐CD133+^) (Fig. 2 *A*). In addition, PC3^DsRed2+^/PC3^DsRed2+GFP‐CD133+^ and DU145^DsRed2+^/DU145^DsRed2+GFP‐CD133+^ cells expressed red fluorescence in the cytosol and at the cell membrane (Fig. 2 *B*). Therefore, we injected mice with PC3^DsRed2+GFP‐CD133+^/DU145^DsRed2+GFP‐CD133+^ (CD133‐overexpressing cells) or PC3^DsRed2+^/DU145^DsRed2+^ (respective control cells).

First, we compared tumor formation following intra‐tibial injection of PC3^DsRed2+GFP‐CD133+^ cells or DU145^DsRed2+GFP‐CD133+^ cells with that after injection of PC3^DsRed2+^ or DU145^DsRed2+^ cells, respectively. When we measured fluorescence generated by GFP and DsRed2 (Fig. 2 *C*), we found that GFP fluorescence in PC3^DsRed2+GFP‐CD133+^ and DU145^DsRed2+GFP‐CD133+^ cells was strongest near the bone lesion and weaker within the tumor mass, suggesting that CD133‐overexpressing PC cells attach to and interact with the bone surface.

### 3.3 CD133 promotes PC3/DU145‐induced osteolytic bone fracture *in vivo*

**Figure 3:**
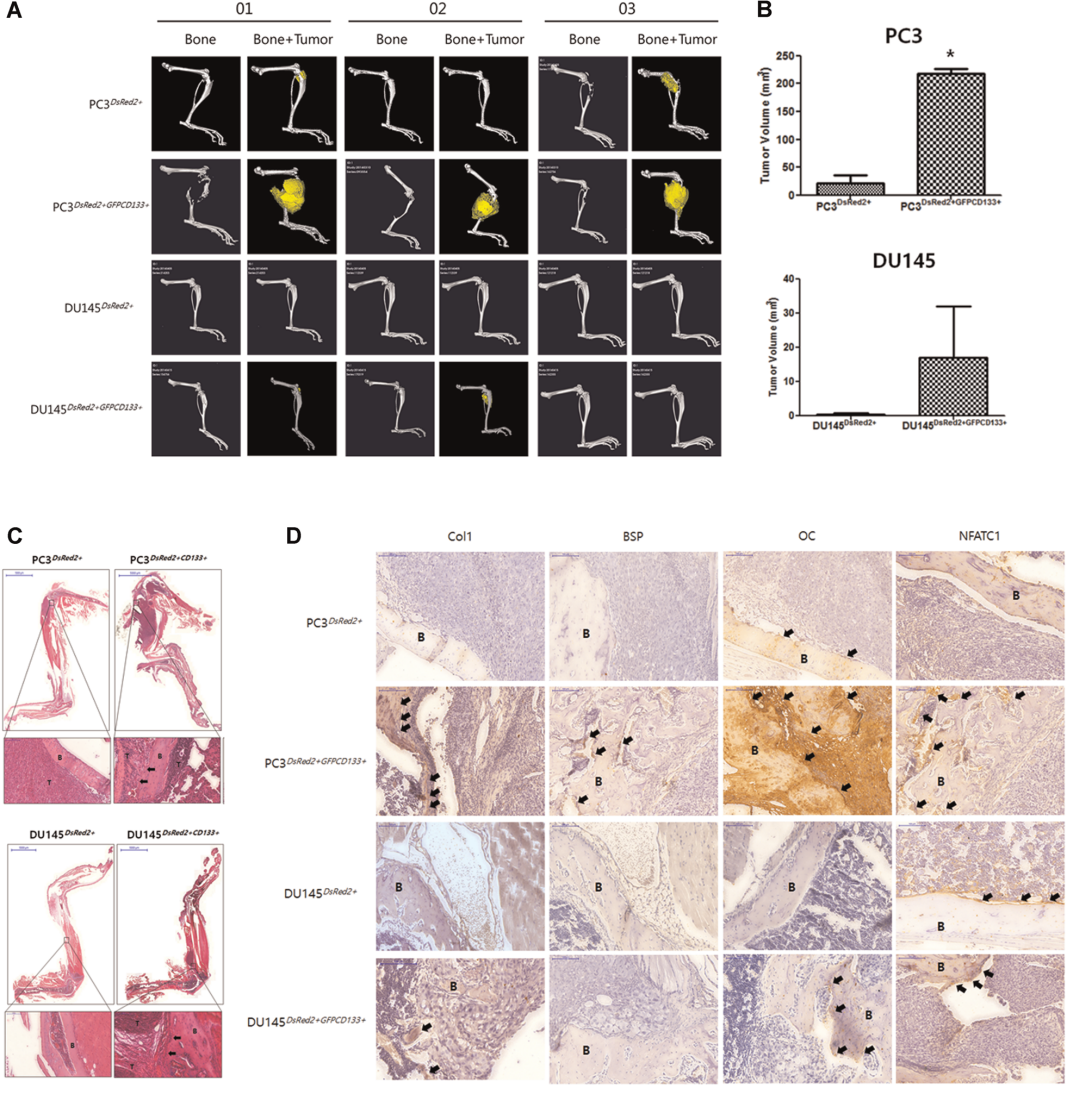
Comparison of tumor‐bearing tibia inoculated with PC3^DsRed2+^/PC3^DsRed2+CD133+^ or DU145^DsRed2+^/DU145^DsRed2+CD133+^ cells. *A*, Representative microcomputed tomography (μCT) images of murine tibiae at 28 days following injection of PC3^DsRed2+^/PC3^DsRed2+CD133+^ and DU145^DsRed2+^/DU145^DsRed2+CD133+^ cells into the bone. Entire tibiae (Bone) were imaged using μCT, and reconstructed 3‐dimensional images were used for visual assessment of bone responses to tumor cells (Bone + Tumor). *B*, Tumor volume was calculated using the ROI tool after 3D rendering of the leg and expressed as the mean ± SD. *C*, Histopathology of mouse tibiae stained with hematoxylin and eosin (H&E) after inoculation of PC3^DsRed2+^/PC3^DsRed2+CD133+^ or DU145^DsRed2+^/DU145^DsRed2+CD133+^ shown at low (upper, 10 × magnification; bar, 5000 μm) and high (lower, 200 × magnification; bar, 100 μm) magnification. B, Bone; T, Tumor mass. *D*, Immunohistochemical analysis of Col1, BSP, OC, and NFATC1 expression in intra‐tibial tumors formed by PC3^DsRed2+^/PC3^DsRed2+CD133+^ or DU145^DsRed2+^/DU145^DsRed2+CD133+^ cells. Negative controls were incubated without the primary antibody (data not shown). High expression of each protein is indicated by a black arrow. Images were taken at 200 × magnification. Bar, 100 μm.

Skeletal tumors clearly caused osteolytic fracture of PC3^DsRed2+GFP‐CD133+^‐injected mouse tibiae, as shown in the 3D micro‐CT scanning image (Fig. 3 *A*). The volume of the PC3^DsRed2+GFP‐CD133+^ bone tumors was much greater (means;200 mm^3^) that that of PC3^DsRed2+^ tumors (25 mm^3^) (Fig. 3 *B*). The volume of DU145^DsRed2+GFP‐CD133+^ tumors was slightly higher (18 mm^3^) than that of respective control tumors.

Table 3 shows that only 30% of animals inoculated with PC3^DsRed2+^ cells developed skeletal tumors (*n* = 10) compared with 100% inoculated with PC3^DsRed2+GFP‐CD133+^ cells. However, only 20% of animals inoculated with DU145^DsRed2+GFP‐CD133+^ cells developed tumors in the tibia; no tumors were observed in mouse tibiae inoculated with DU145^DsRed2+^ cells. Taken together, these data suggest that ectopic overexpression of CD133 leads to a significant increase in bone tumor volume in vivo, and that increased CD133 expression may increase the risk of metastasis.

**Table 3:** Gene Primer Sequences

Gene name	Upstream primer (5′‐3′)	Downstream primer (3′‐5′)
***OCN***	CTC CTT ACC CGG ATC CCC TG	GTA GAA GCG CTG GTA GGC GT
***GAPDH***	TGG AAT CCA CTG GCG TCT TC	GGT TCA CGC CCA TCA CAA AC

Next, we performed H&E staining to examine the histologic features of skeletal tumors formed by PC3^DsRed2+^/PC3^DsRed2+GFP‐CD133+^ and DU145^DsRed2+^/DU145^DsRed2+GFP‐CD133+^ cells inoculated into mouse tibiae. As shown in Fig. 3 *C*, gross examination of excised tibiae revealed that in general the tumor mass occupied the primary spongiosum (trabecular epiphysis) and displaced the bone marrow cells; this was not the case in tibiae inoculated with PC3^DsRed2+^ or DU145^DsRed2+^ cells. In particular, there was an apparent margin between bone and tumor in PC3^DsRed2+^‐inoculated tibiae; however, we noted osteoblastic or osteolytic features in tibiae inoculated with PC3^DsRed2+GFP‐CD133+^.

Therefore, we next examined expression of bone‐related proteins by PC3^DsRed2+^/PC3^DsRed2+GFP‐CD133+^ and DU145^DsRed2+^/DU145^DsRed2+GFP‐CD133+^ cells in mouse tibiae (Fig. 3 *D*). Clinically, PC bone metastases show a predominantly osteoblastic phenotype mixed with osteolytic components; however, inoculation of PC cells induces mainly osteolytic bone fractures. Therefore, to assess whether overexpression of CD133 affects the osteosclerotic/osteolytic phenotype of PC3/DU145 cells, we examined expression of collagen type I (Col1), bone sialoprotein (BSP), osteocalcin (OC), and Nuclear factor of activated T‐cells, cytoplasmic 1 (NFATC1) by PC3^DsRed2+^/PC3^DsRed2+GFP‐CD133+^ and DU145^DsRed2+^/DU145^DsRed2+GFP‐CD133+^ cells inoculated into mouse tibiae. Positive expression of Col1, OC, and NFATC1 was observed in the trabecular epiphysis region of bone inoculated with PC3^DsRed2+GFP‐CD133+^ cells. In addition, marked expression of OC was detected in the trabecular epiphysis region of PC3^DsRed2+GFP‐CD133+^‐inoculated tibiae. These observations suggest that ectopic overexpression of CD133 leads to osteosclerotic and osteolytic activation of bone adjacent to the tumor, particularly in bone injected with PC3 cells, and that CD133 expression is associated with metastasis of PC to bone.

### 3.4 Expression of osteosclerotic/osteolytic‐related cytokines and their role in subsequent molecular events

**Figure 4:**
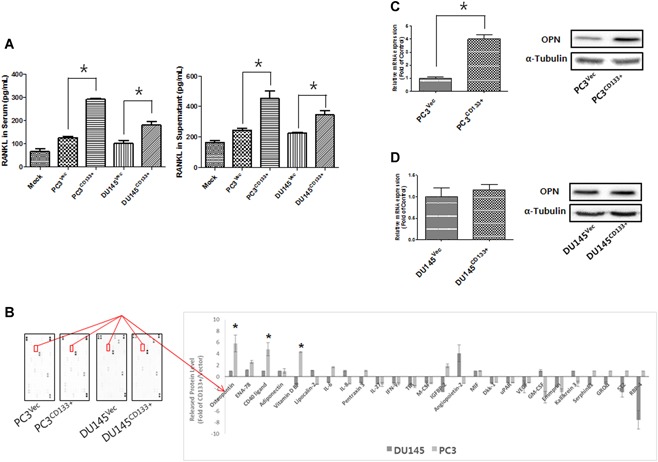
Components associated with osteogenesis and osteolysis. *A*, Concentration of RANKL in cell culture supernatant or serum of mice inoculated with prostate cancer cells. Bar graphs show the mean ± standard deviation (SD). *, *P* < 0.05. *B*, Cytokine profiling of PC3^Vec^/PC3^CD133+^ and DU145^Vec^/DU145^CD133+^ cells. Culture supernatant from each cell line was harvested after 24 hours and assayed using a cytokine profile array kit. The bar represents the mean ratio ± SD from two experiments. Significant differences were seen at *P < 0.05 (compared with PC3^Vec^/PC3^CD133+^ and DU145^Vec^/DU145^CD133+^ cells). *C*, Expression of OPN mRNA and protein by PC3^Vec^ and PC3^CD133+^ cells was measured by real‐time PCR and Western blot analysis, respectively. α‐tubulin was used as a loading control. *, *P* < 0.05. *D* OPN mRNA and protein expression by DU145^Vec^ and DU145^CD133+^ cells was characterized by real‐time PCR and Western blot analysis, respectively.

To assess the osteolytic activity of PC cell lines, we measured the amount of RANKL released into the culture supernatant or mouse serum by PC3^Vec^/PC3^CD133+^ and DU145^Vec^/DU145^CD133+^ cells. We found that ectopic overexpression of CD133 increased the amount of RANKL in both cell culture supernatant and serum. This suggests that overexpression of CD133 by PC cell lines contributes to osteolytic bone fracture upon metastasis to bone (Fig. 4 *A*).

Next, we tried to identify other molecules that play a critical role in development of osteosclerotic or osteolytic characteristics by screening cytokines using a proteome array cytokine kit (Fig. 4 *B*). Overexpression of CD133 by PC3 cells led to a marked increase in expression of CD40 ligand, vitamin D‐binding protein, and (especially) osteopontin (OPN); these increases were greater than those observed for DU145 cells. Indeed, levels of OPN mRNA and protein were higher in PC3^CD133+^ cells than in PC3^Vec^ cells (Fig. 4 *C*). However, overexpression of CD133 did not increase the amount of OPN mRNA and protein in DU145 cells (Fig. 4 *D*).

### 3.5 Effect of OPN silencing in PC3^Vec^ /PC3^CD133+^ cells and in murine BMSCs on bone mineralization and ALP activity

**Figure 5:**
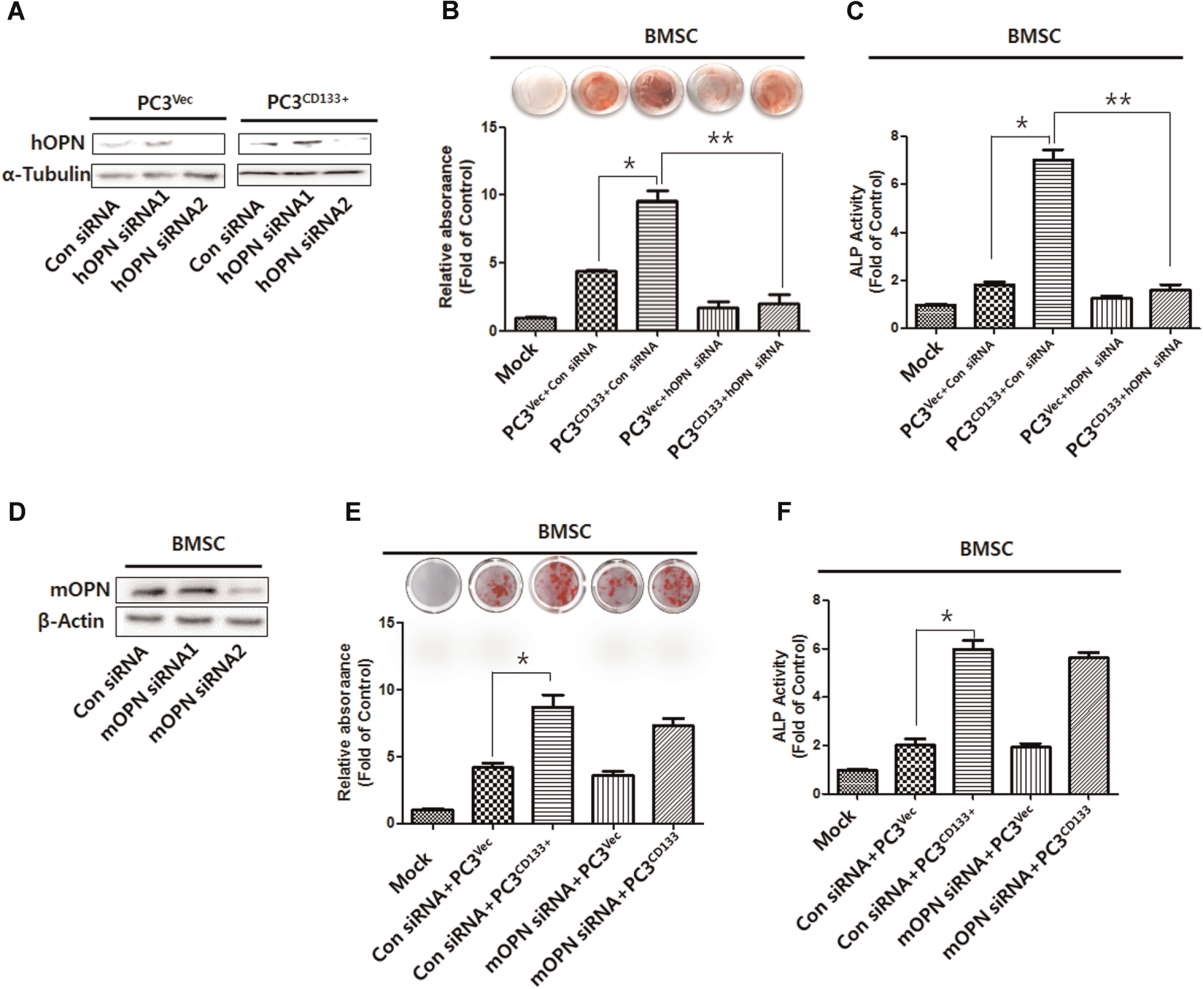
Effect of knocking down OPN on bone mineralization and ALP activity. *A*, Expression of hOPN protein in PC3^Vec^ and PC3^CD133+^ cells transfected with control siRNA, hOPN siRNA1, or hOPN siRNA2 was assessed by Western blot analysis. α‐tubulin was used as a loading control. Similar results were obtained in three separate experiments. *, *P* < 0.05. *B*, Mineralized BMSCs were cultured in Mock medium, conditioned medium (CM) from control siRNA‐treated PC3^Vec^ cells, CM from control siRNA‐treated PC3^CD133+^ cells, CM from hOPN siRNA‐treated PC3^Vec^ cells, or CM from hOPN siRNA‐treated PC3^CD133+^ cells. BMSCs were then stained with 2% Alizarin Red S (AR‐S) at 28 days. In addition, the AR‐S concentration was assessed by measuring absorbance at 540 nm. Bar graphs show the mean ± the standard deviation (SD). *, *P* < 0.05. *C*, ALP activity of BMSCs. All data represent the mean ± SD (*n* = 3). *D*, Expression of mOPN protein by BMSCs treated with control siRNA, mOPN siRNA1, or mOPN siRNA2 was assessed by Western blot analysis. α‐tubulin was used as a loading control. *E* Mineralized BMSCs were cultured in Mock medium, or with control siRNA and CM from PC3^Vec^ cells, control siRNA and CM from PC3^CD133+^ cells, mOPN siRNA and CM from PC3^Vec^ cells, or mOPN siRNA and CM from PC3^CD133+^ cells. BMSCs were then stained with 2% Alizarin Red S (AR‐S) at 28 days. *F* ALP activity of BMSCs. All data represent the mean ± SD (*n* = 3).

To investigate the effect of OPN on osteoblastic differentiation of osteoblast progenitor cells upon metastasis of PC cells, PC3^Vec^ or PC3^CD133+^ cells were transfected with control siRNA or with two different siRNAs targeting human (h)OPN. Figure. 5 *A* shows that silencing of OPN by siRNA2 led to marked downregulation of OPN expression by both PC3^Vec^ and PC3^CD133+^ cells. Therefore, we used hOPN siRNA2 for further studies. To examine the effect of hOPN on PC‐induced osteosclerosis, we cultured bone marrow stromal/pre‐osteoblast cells (BMSCs) from mouse tibia for 28 days in conditioned medium (CM) from PC3^Vec^ cells or PC3^CD133+^ cells treated with control siRNA or hOPN siRNA2, respectively. BMSCs exposed to CM from PC3^CD133+^ cells transfected with control siRNA showed strongly staining when exposed to Alizarin red (Fig. 5 *B*). However, exposure to CM from PC3^CD133+^ cells transfected with hOPN siRNA2 led to a reduction in the amount of mineralized matrix stained with Alizarin red. CM from PC3^Vec^ and PC3^CD133+^ cells harboring control siRNA induced a significant increase in ALP activity by BMSCs at Day 7 (Fig. 5 *C*); however, this was reversed by exposure to CM from PC3^CD133+^ cells harboring silenced hOPN.

To determine the autocrine effects of OPN on osteoblastic differentiation during metastasis of CD133 + PC cells, BMSCs were transfected with control siRNA or two species of siRNA specific for mouse osteopontin (mOPN). Figure. 5 *D* shows that mOPN siRNA2 reduced OPN expression by BMSC cells. Therefore, BMSCs were transfected with mOPN siRNA2 in subsequent experiments. Control siRNA‐ and mOPN siRNA‐treated BMSCs showed similar levels of Alizarin red‐stained mineralized matrix and ALP activity upon exposure to CM from both of PC3^Vec^ and PC3^CD133+^ cells (Fig. 5 *E, F*). These results suggest that mOPN from BMSC does not have autocrine effects on osteoblastic differentiation by stimulating CD133 + PC cells.

### 3.6 Effect of OPN‐silenced PC3^CD133+^ cells on expression of OC, Runx2, and OSX

**Figure 6:**
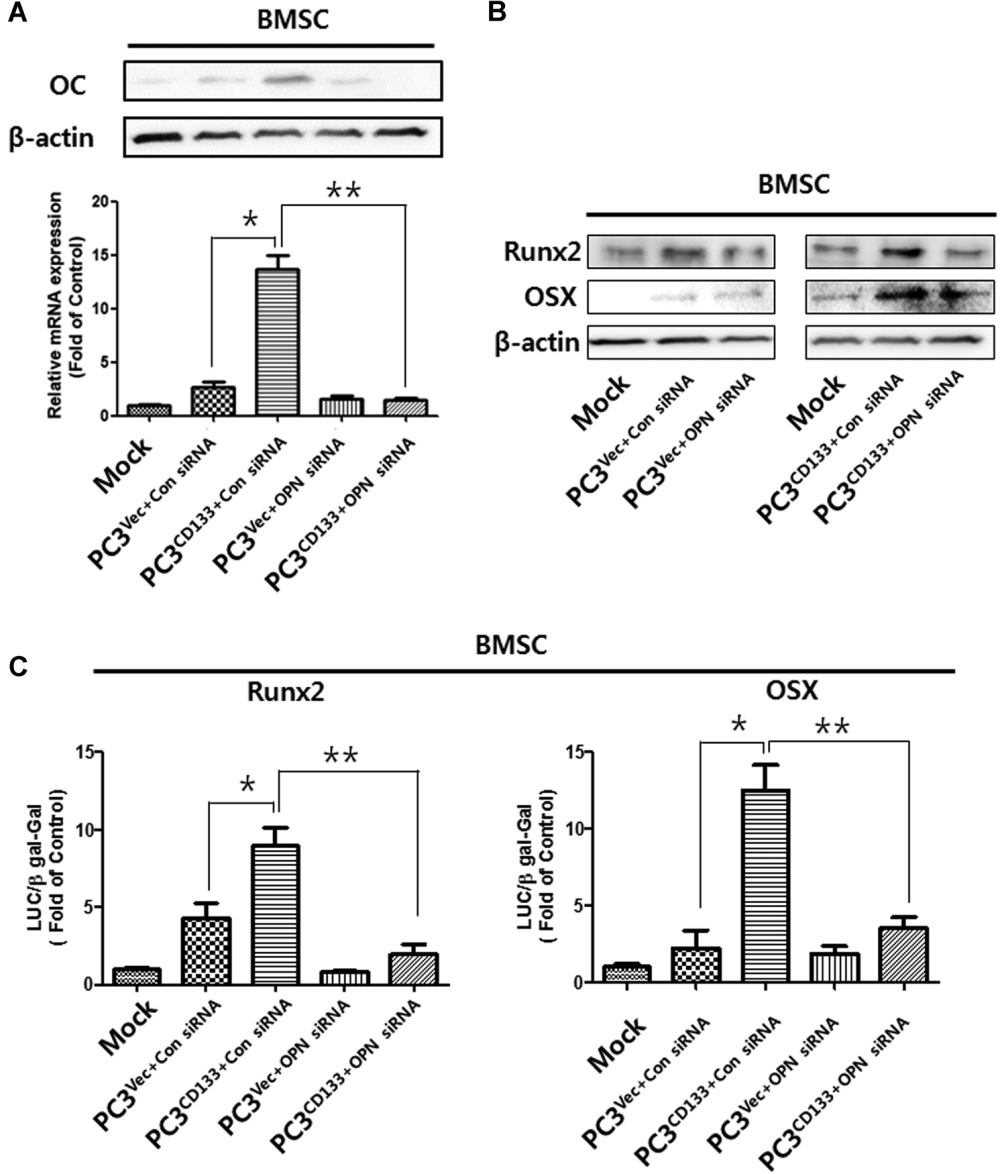
Effect of knocking down hOPN on expression of OC, Runx2, and OSX. *A* Expression of OC mRNA and protein in BMSCs cultured in Mock medium, conditioned medium (CM) from control siRNA‐treated PC3^Vec^ cells, CM from control siRNA‐treated PC3^CD133+^ cells, CM from hOPN siRNA‐treated PC3^Vec^ cells, or CM from hOPN siRNA‐treated PC3^CD133+^ cells. BMSCs were then analyzed by Western blot analysis and real‐time PCR at 28 days. β‐actin was used as a loading control. Similar results were obtained in three separate experiments. *, *P* < 0.05. *B* Expression of Runx2 and OSX proteins in BMSCs after 7 days of culture in Mock medium/CM from control siRNA‐treated PC3^Vec^ cells/CM from control siRNA‐treated PC3^CD133+^ cells, or in Mock medium/CM from hOPN siRNA‐treated PC3^Vec^ cells/CM from hOPN siRNA‐treated PC3^CD133+^ cells. *C* BMSCs were transiently transfected with a pRunx2‐Luc or pOSX‐Luc reporter gene and then treated with the medium described in (a) for 7 days. Luciferase activity was measured as described in the “Materials and methods” section. Results are expressed as mean ± SD of triplicate tests. * and **, *P* < 0.05.

BMSCs cultured with PC3^CD133+^ cells transfected with control siRNA showed a significant increase in expression of OC mRNA and protein (Fig. 6 *A*). However, PC3^CD133+^ cells transfected with hOPN siRNA showed decreased expression of OC mRNA and protein. To determine the inhibitory effect of hOPN silencing on Runx‐related transcription factor 2 (Runx2)‐ and osterix (OSX)‐related pathways, both of which are master transcription factors for controlling osteoblast differentiation, we cultured BMSCs in CM from OPN siRNA‐treated PC3^CD133+^ cells and measured expression of Runx2 and OSX protein by Western blot analysis (Fig. 6 *B*). Treatment with CM from OPN siRNA‐treated PC3^CD133+^ cells led to a decrease in expression of Runx2 and OSX by BMSCs. In addition, to investigate the effects of OPN silencing on Runx2 and OSX activity, BMSC cells were transiently transfected with a pRunx2‐Luc or pOSX‐Luc vector. Exposure to CM from PC3^CD133+^ induced a significant increase in OSX activity in BMSCs (Fig. 6 *C*). However, CM from silenced hOPN PC3^CD133+^ cells decreased OSX activity in BMSCs. These results are consistent with the notion that hOPN (or the OPN‐stimulated signal transduction pathway) has a direct effect on osteoblastic differentiation via OSX activation by CD133 + PC cells.

## Discussions

Bone is a common site of distant metastasis in PC; indeed, the association between bone and metastatic cancer is an important driver of this site‐specific manifestation of PC progression.^26^ During the last decades, research has identified CSCs as central players in cancer metastasis. CSCs are a small population of cells responsible for initiation of tumor burden and therapeutic refractoriness.^27^ Acquisition of self‐renewal capacity and stemness properties at the time of tumorigenesis is an essential prerequisite for further accumulation of oncogenic transformations and eventual development of cancer.^28^ Here, we sought to identify CSC‐like cells by inducing ectopic overexpression of CD133 in two PC cell lines (PC3 and DU145). It led the genetic changes in PC cells, culminating in altered expression of several factors related to stemness, including OCT4, ALDHA1, and NANOG, resulting in increased colony‐forming ability and ALDH activity. CD133‐expressing cancer cells with a stem cell‐like phenotype are responsible for tumor recurrence.^29^ CD133 is also a common marker of stemness and expressed by normal tissue‐resident and hematopoietic stem cells; however, it is no longer detectable after differentiation. Likewise, cancer cells stop expressing CD133 upon differentiation.^30^ Colon, lung, and hematopoietic CSCs are isolated by cell sorting based on expression of CD133, although the function of CD133 remains unclear.^31^ In vitro, these cells grow indefinitely as spheres and acquire tumorigenicity in vivo. Here, we found that overexpression of CD133 was associated with stemness and migration of PC cells.

So, is it possible that the CSC properties of PC cells are responsible directly for bone metastasis? To answer this question, we cotransfected PC3 or DU145 PC cells with CD133 and fluorescent proteins DsRed2. These cells (along with their respective controls) were then inoculated into the right tibia of athymic nu/mice. Considering the hypothesis that CSCs give rise to both CSCs and non‐CSCs, we expected to find CD133 + and CD133‐ cells. Hongo et al reported that CD133+ cells can give rise to CD133‐ cells, but not vice versa, indicating that CD133 is responsible for stemness.^32^ CSCs expand through asymmetric cell division, the result of which is two daughter cell populations; one is similar to the mother cell (retaining stem cell properties) while the other is committed to another specific line of differentiation.^33^ These characteristics of CSCs are responsible for tumor heterogeneity, meaning that expression of CD133 varies widely within a single cancer cell line or a single tumor.^34^ Our findings indicate that CD133 is likely to be one of perhaps several stem cell‐related molecules involved in PC metastasis to bone. Radiologic and histologic findings suggested that CD133+ PC3 cells generated larger and more aggressive tumors than wild‐type cells. In addition, we conducted a comparative analysis of the biologic characteristics of CD133 + and wild‐type PC cells. The results showed that PC3^DsRed2+GFP‐CD133+^ cells generated GFP and DsRed2 fluorescence when they formed a tumor mass in the mouse tibia; moreover, the GFP signal was observed in a region attached to bone. Thus, we believe that attachment of CD133 + cells, which have stemness properties, to bone causes a high rate of bone fracture and osteolysis. Detailed histomorphometric analyses revealed that fast‐growing intra‐tibial PC3^DsRed2+GFP‐CD133+^/DU145^DsRed2+GFP‐CD133+^tumors formed heterogeneous lesions in bone, thereby causing higher levels of lysis than PC3^DsRed2+^/DU145^DsRed2+^ mock tumors at the same time points. The most apparent feature of PC3^DsRed2+GFP‐CD133+^/DU145^DsRed2+GFP‐CD133+^intra‐tibial tumors was a marked increase in lysis of local bone structures. In particular, micro‐CT and histologic analyses revealed that PC3^DsRed2+GFP‐CD133+^ tumors almost completely destroyed the tibial bone architecture after 3 weeks post‐cancer cell inoculation, whereas damage caused by PC3^DsRed2+^ was much less severe. NFATC1 is an osteoclastic marker, expression of which is observed in highly active osteoclast cells. Here, staining for NFATC1 revealed a high number of osteoclasts at the intra‐tibial tumor/bone interface; in addition, the amount of RANKL in cell culture supernatants and mouse serum increased. This may explain the massive local net bone lysis caused by PC3^CD133+^/DU145^CD133+^ intra‐tibial tumors.

A previous study shows that PC3 cells generate a predominantly lytic response within bone; however, actual deposition of new bone was also observed.^26^ Similarly, clinical studies show that in most PC patients bone lesions involve both bone destruction and bone formation at metastatic foci.^35^, ^36^, ^37^ The tropism of PC cells to bone suggests that they interact preferentially with specific cells in the bone microenvironment; the most likely candidates are osteoblasts.^38^ Radiologic and histomorphometric evidence indicates that sites of PC metastases in bone often show microscopic evidence of increased bone production, including a greater osteoid surface, an increased osteoid volume, and higher rates of mineralization.^4^ The present histological findings are consistent with experimental evidence of increased expression of osteoblastic markers such as Col1, BSP, and (especially) OC in PC3^DsRed2+GFP‐CD133+^‐inoculated murine tibiae, especially, suggesting that PC (characterized by increased CSC‐like properties) induces bone production via an overall increase in bone remodeling. In particular, undifferentiated carcinomas are more aggressive and metastatic; thus increased CSC‐like properties have an effect on osteoblastic differentiation of BMSCs.^39^ In the case of PC, it appears that induction of osteoblast‐mediated mineralization eventually outweighs the increase in osteoclast resorption, resulting in formation of osteoblastic lesions.^26^

**Figure 7:**
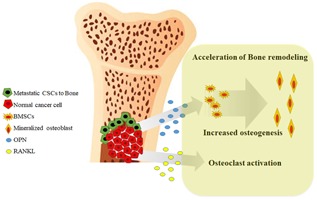
Proposed role of OPN during metastasis of prostate cancer to bone. Metastatic CSCs settle in the bone marrow, where they secrete RANKL and OPN, thereby inducing BMSCs to differentiate into mineralized osteocytes. CSCs then influence bone remodeling and cause osteolytic bone fracture.

Various regulatory factors play a role in proper development and maintenance of bone structure during cancer metastasis to bone. So, which factors play a critical role in CD133+PC cell‐induced bone remodeling? Here, we found that PC3^CD133+^ cells showed higher expression of OPN than DU145^CD133+^ cells, suggesting that OPN elicits essential biological functions and may play a critical role of osteosclerosis during PC metastasis to bone.^40^, ^41^ OPN, also called secreted phosphoprotein 1 (SPP1), was initially identified as an extracellular matrix protein that inhibited formation and growth of hydroxyapatite crystals within the bone matrix and other organs.^42^ Osteoblasts are the major source of OPN in bone tissue. Generally, OPN is recognized as a middle‐stage marker of osteoblastic differentiation,^43^ suggesting that it plays an essential role in regulating bone metabolism. In addition, OPN is overexpressed by various cancers and is regarded as a novel cancer marker. OPN is secreted by malignant tumor cells and promotes self‐adhesion, invasion, metastasis, neovascularization, and formation of new tumor tissue. The findings presented herein indicate that OPN affects osteoblast physiology in an endocrine manner by increasing the ALP activity and OC expression during PC3^CD133+^‐induced BMSC differentiation. In addition, we found that a lack of OPN in CM led to reduced ALP activity and OC expression by BMSCs, resulting in a decrease in Alizarin red‐positive mineral density. These changes suggest that OPN upregulates osteoblastic differentiation and influences the extent of tissue mineralization. This is consistent with previous studies pointing to a role for increased OPN deposition in the pathologies underlying mineralization and bone metastasis of breast cancer cells.^44^, ^45^ In addition, Runx2 and OSX (osteogenesis‐specific transcription factors) are expressed during osteoblastic differentiation of BMSCs.^46^, ^47^ Runx2 is required for differentiation of mesenchymal cells into pre‐osteoblasts. As a molecule downstream of Runx2, Osx is required for differentiation of pre‐osteoblasts into mature osteoblasts. In particular, Osx is necessary for bone formation and mineralization in vivo, and mature functioning osteoblasts strongly express later osteoblast marker genes such as OC and BSP in response to OSX signals.^48^ Here, CM from PC3^CD133+^ cells induced a significant increase in OSX activity in BMSCs; however, silencing of OPN decreased OSX activity in BMSCs. We identified a close relationship between increased expression of OC in the PC3^CD133+^‐inoculated mouse tibia and increased OSX expression and activity; also, had a direct effect on osteoblastic differentiation via activation of OSX by CD133 + PC cells. The data demonstrate that CSC‐like properties play a major role in bone remodeling in marrow due to increased release of OPN. Bone metastasis of PC resulted in mixed, heterogeneous osteoblastic and osteolytic lesions. Osteoblastic lesions are characterized by excess deposition of new bone, whereas osteolytic lesions are characterized by destruction of bone. These two types of PC metastasis disrupt the bone microenvironment. In particular, CSC‐like properties accelerate secretion of factors that stimulate proliferation and differentiation of osteoprogenitor cells, or induce osteoclast precursors to differentiate into mature osteoclasts in the tumor environment. Here, we demonstrated that release of OPN from CSC candidate cells triggered bone formation, suggesting that PC bone metastases initiate osteoblastic lesions. OPN acts as a stimulator of osteogenesis by regulating OSX, which can function in an autocrine, paracrine, or endocrine manner (Fig. 7).

In light of significant advances in our knowledge of metastasis and stem cell biology, we propose a CSC‐based model of osteosclerotic remodeling during bone metastasis of PC. The findings indicate that CD133 plays a central role in regulating CSCs in PC, resulting in acquisition and maintenance of osteolytic and osteosclerotic characteristics. Furthermore, this hypothesis substantiates the role of stem cells in cancer metastasis to bone. Therefore, the results should facilitate development of a novel classification system and provide new avenues to developing therapeutic strategies for bone metastasis.

## Disclosures

The authors declare that they have no conflict of interest.
